# Negative association of composite dietary antioxidant index and peripheral artery disease in US participants: A cross-sectional study

**DOI:** 10.1097/MD.0000000000050586

**Published:** 2026-09-11

**Authors:** Qiang Liu, Xing Wu, Jun Yan, Yigang He, Yun Wang, Jianjun Shi

**Affiliations:** a Department of Cardiovascular Surgery, Taiyuan City Central Hospital, Taiyuan, Shanxi, China; b Department of Cardiovascular Surgery, The Ninth Clinical College Affiliated to Shanxi Medical University, Taiyuan, Shanxi, China; c Department of Nephrology, Taiyuan City Central Hospital, Taiyuan, Shanxi, China; d Department of Nephrology, The Ninth Clinical College Affiliated to Shanxi Medical University, Taiyuan, Shanxi, China.

**Keywords:** ankle-brachial index, composite dietary antioxidant index, cross-sectional study, National Health and Nutrition Examination Survey, peripheral artery disease

## Abstract

The association between the composite dietary antioxidant index (CDAI) and peripheral artery disease (PAD) remains inadequately explored. Elucidating this link is crucial for developing targeted nutritional strategies to mitigate PAD risk at both individual and public health levels. This analysis sought to evaluate the relationship between CDAI scores and the prevalence of PAD. We conducted a cross-sectional analysis utilizing data from the National Health and Nutrition Examination Survey (1999–2004). Participant demographics (age, sex, race, education, marital status, income-to-poverty ratio) and health-related factors (physical activity, body mass index, smoking status, total cholesterol, C-reactive protein, glycosylated hemoglobin, and histories of cardiovascular disease, hypertension, and diabetes) were collected. The association was examined using logistic regression, smoothed curve fitting, and interaction analyses. The study included 6018 individuals, with a PAD prevalence of 5.9% (n = 358). After comprehensive covariate adjustment, a significant inverse association between CDAI and PAD prevalence was observed (odds ratio [OR] = 0.96, 95% confidence interval [CI]: 0.92–1.00). When CDAI was analyzed as tertiles, participants in the middle tertile (T2) showed significantly lower odds of PAD compared to the lowest tertile (T1) (OR = 0.74; 95% CI = 0.56–0.98). The highest tertile (T3) also exhibited reduced odds, though not statistically significant (OR = 0.93; 95% CI = 0.69–1.24). Subgroup analyses consistently demonstrated this inverse trend, with no significant interaction effects detected (all *P* for interaction > .05). Our findings indicate that a higher CDAI is independently associated with a lower prevalence of PAD in United States adults. Future research is warranted to confirm these results and explore the underlying mechanisms.

## 1. Introduction

Peripheral artery disease (PAD) is a prevalent atherosclerotic condition marked by the narrowing of arteries in the lower limbs due to plaque buildup.^[1]^ It represents a significant and growing public health challenge worldwide, affecting over 200 million individuals globally and more than 8.5 million adults aged 40 and above in the United States (US), with comparable prevalence between sexes.^[2,3]^ The considerable economic and health consequences of PAD underscore the urgency of identifying modifiable risk factors. Pathophysiological studies have emphasized the roles of inflammatory markers, endothelial dysfunction, and oxidative stress in PAD development.^[4–9]^ Although smoking, diabetes, hypertension, and dyslipidemia are established risk factors,^[10]^ the influence of dietary patterns, particularly overall antioxidant capacity, is less documented. Enhancing our understanding of dietary prevention is therefore essential for improving PAD management.

As a primary indicator of systemic atherosclerosis, PAD involves the narrowing of arteries supplying the legs.^[11]^ Both experimental and epidemiological evidence highlight oxidative stress as a critical driver in the initiation and progression of atherosclerosis.^[12–15]^ Dietary intake of exogenous antioxidants plays a key role in supporting the body’s endogenous defense systems.^[16]^ Prior studies have suggested that increased consumption of specific antioxidants, including carotenoids and vitamins C and E, may lower PAD risk.^[17–19]^ However, these investigations have typically focused on single nutrients, leaving the collective effect of overall dietary antioxidant intake on PAD unclear.^[20–22]^ To address this gap, the composite dietary antioxidant index (CDAI) was developed to quantify the total antioxidant potential from daily food intake.^[23]^ The relationship between CDAI and PAD in the adult population, however, has not been established. This study, therefore, aimed to examine the association of CDAI with PAD and discuss its potential implications, using a retrospective cross-sectional design with data from 6018 US residents.

## 2. Materials and methods

### 2.1. Study population

This cross-sectional study analyzed data from 6018 participants in the US National Health and Nutrition Examination Survey (NHANES, 1999–2004 cycles). NHANES employs a complex, multistage sampling design to collect nationally representative data on health and nutritional status through interviews, physical examinations, and laboratory tests.^[24]^ The National Center for Health Statistics ethics review board approved the survey protocol, and all participants provided written informed consent.^[25]^ Publicly accessible data can be obtained at http://www.cdc.gov/nchs/nhanes.htm. From an initial pool of 31,126 interviewed individuals, we excluded those under 40 years (n = 21,156) due to the unavailability of ankle-brachial index (ABI) measurements for this age group. Further exclusions included missing bilateral ABI data (n = 2399), ABI values > 1.4 (n = 73),^[26,27]^ insufficient dietary information (n = 169), and missing data on key covariates, resulting in the final analytic sample of 6018 participants. As this study was a secondary analysis of de-identified, publicly available NHANES data, ethical review and approval were waived, and no additional institutional review board approval was required.

### 2.2. Assessment of PAD

ABI measurements were performed on participants aged 40 years and older following a standardized protocol. After a 5-minute rest period, systolic blood pressure (SBP) was measured in the right arm and both ankles. If the right arm measurement was unavailable, the left arm was used instead. For individuals aged 40 to 59, SBP was measured twice; for those 60 and above, it was measured once. The ABI for each leg was calculated by dividing the mean ankle SBP by the mean arm SBP. PAD was defined as an ABI value of < 0.9 in either leg.^[28,29]^

### 2.3. Calculation of CDAI

Dietary data were derived from the first-day 24-hour dietary recall interviews in NHANES, conducted using the computer-assisted dietary interview system. This method captures detailed information on all foods and beverages consumed in the previous 24 hours, including type, amount, time, and occasion. The United States Department of Agriculture’s Automated Multiple Pass Method was employed to enhance recall accuracy. While 24-hour recalls have inherent limitations, they provide more precise data on specific food items and quantities compared to food frequency questionnaires.^[30,31]^ The CDAI, a composite measure reflecting the intake of 6 dietary antioxidants (selenium, magnesium, zinc, vitamin A, vitamin C, and vitamin E), was calculated for each participant.^[23]^ The intake of each component was standardized by subtracting the global mean and dividing by the global standard deviation. The CDAI score was then computed as the sum of these standardized values, as per the following formula^[32–34]^:


$$CDAI=\overset{n=6}{\underset{i=1}{\sum }}\;\;\;\frac{Individual\;\;\;Intake-Mean}{SD}$$

### 2.4. Covariate assessment

Sociodemographic and lifestyle information was collected through standardized questionnaires. Physical examinations and laboratory tests provided data on body mass index (BMI), blood pressure, total cholesterol, C-reactive protein (CRP), and glycated hemoglobin (hemoglobin A1c [HbA1c]). Covariates were selected based on previous literature^[13,22,30,31,35–37]^ and included age, sex, race/ethnicity, education level, marital status (MS), poverty-to-income ratio (PIR), smoking status, physical activity, BMI, and laboratory parameters. We also accounted for self-reported physician diagnoses of diabetes, hypertension, and cardiovascular disease (CVD, encompassing congestive heart failure, coronary heart disease, angina, myocardial infarction, or stroke). PIR was categorized as low (≤ 1.3), medium (1.3–3.5), or high (> 3.5). Smoking status was defined as never, former, or current smoker. Physical activity levels were classified as sedentary (no moderate or vigorous activity), moderate (moderate but no vigorous activity), or vigorous (any vigorous activity). Hypertension was defined as average blood pressure ≥ 140/90 mm Hg, current use of antihypertensive medication, or prior diagnosis. Diabetes was defined by fasting glucose > 7 mmol/L, random glucose ≥ 11.1 mmol/L, HbA1c ≥ 6.5%, use of hypoglycemic agents, or previous diagnosis. BMI was calculated as weight in kilograms divided by height in meters squared.

### 2.5. Statistical analysis

We performed a secondary analysis of the NHANES dataset. Categorical variables are expressed as frequencies (percentages), and continuous variables as means (± standard deviation) or medians (interquartile range) based on their distribution. Group differences were assessed using 1-way analysis of variance, Kruskal-Wallis tests, or chi-square tests, as appropriate. Logistic regression models were used to estimate odds ratios (ORs) and 95% confidence intervals (CIs) for the association between CDAI and PAD. Confounders were selected a priori based on clinical relevance and prior studies. Four sequential models were constructed: Model 1 (unadjusted); Model 2 (adjusted for age, sex, race, education, MS, PIR, and physical activity); Model 3 (further adjusted for BMI, total cholesterol, CRP, and HbA1c); and Model 4 (fully adjusted for all covariates, including hypertension, diabetes, and CVD history). We also conducted subgroup analyses and interaction tests using likelihood ratio tests. Notably, this analysis did not employ NHANES sampling weights, and thus estimates are unweighted. All analyses were conducted using R Statistical Software (version 4.2.2; http://www.freestatistics.net) and the Free Statistics Analysis Platform (version 1.9).

## 3. Results

### 3.1. Baseline characteristics

**Figure 1.**
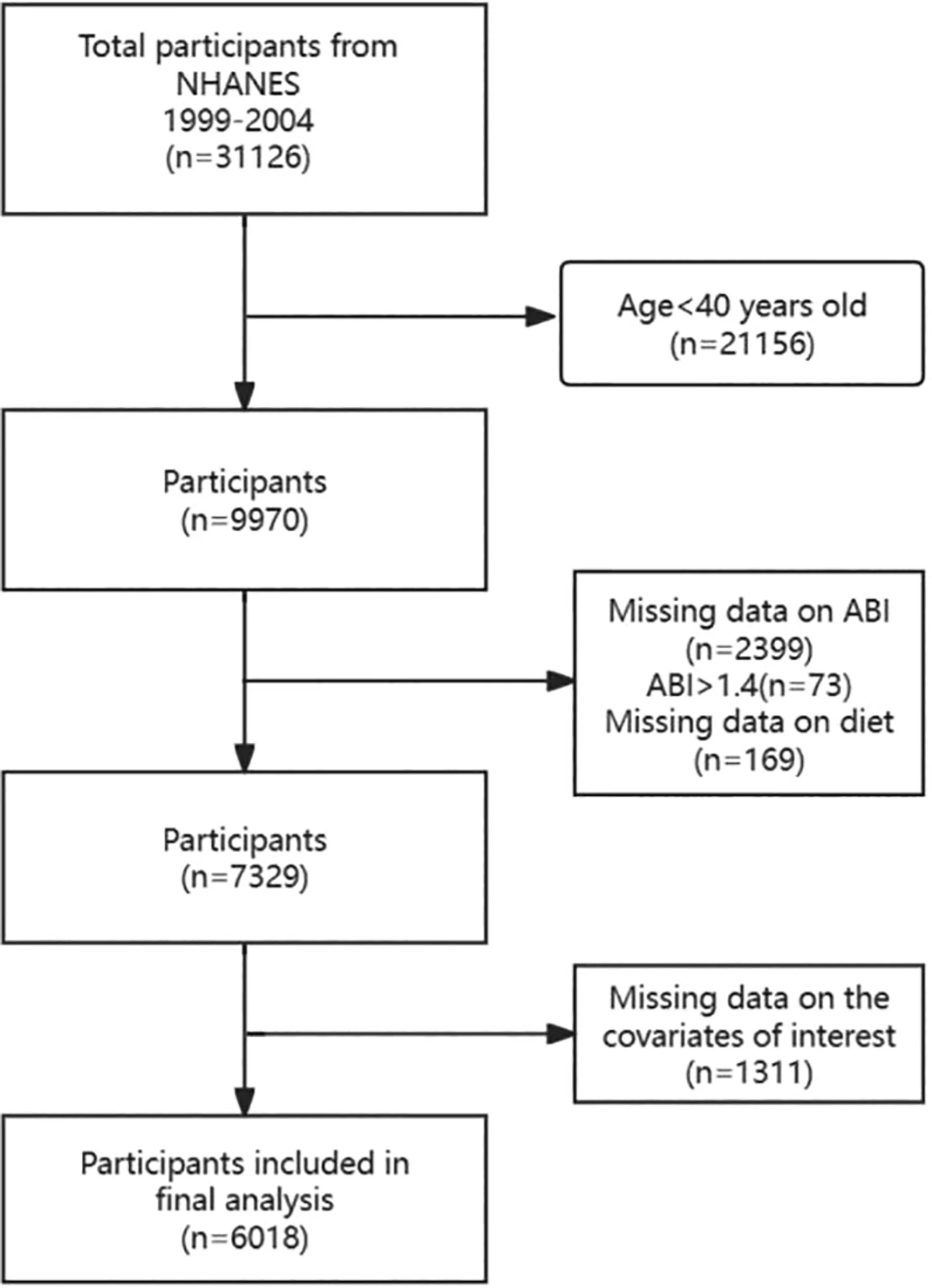
The flow chart of the study. ABI = ankle-brachial index, n = number of participants, NHANES = National Health and Nutrition Examination Survey.

From 31,126 initial interviewees, 6018 eligible participants were included in the final analysis (Fig. 1). The overall prevalence of PAD was 5.9%. Table 1 summarizes participant characteristics across CDAI tertiles. Significant differences (*P* < .05) were observed for most variables, including sex, age, education, race, MS, PIR, physical activity, HbA1c, CRP, smoking status, and histories of hypertension, CVD, and diabetes. Participants in the highest CDAI tertile (T3) were generally younger (mean age 58.1 vs 61.3 years in T1), more likely to be male (66.4% vs 37.9%), non-Hispanic White (60.7% vs 48%), and have higher educational attainment and income. They also reported more vigorous physical activity, had lower CRP levels, and lower rates of hypertension, CVD, and current smoking. No significant differences were found for BMI (*P* = .846) or total cholesterol (*P* = .249). The prevalence of PAD decreased across increasing CDAI tertiles (T1: 8.2%, T2: 5.0%, T3: 4.7%; *P* < .001).

**Table 1:** Baseline characteristics stratified by CDAI tertiles (T).

Variables	Total	CDAI			*P* value
	(n = 6018)	T1 (n = 2006)	T2 (n = 2006)	T3 (n = 2006)	
Age (yrs)	59.9 ± 13.0	61.3 ± 12.9	60.2 ± 13.1	58.1 ± 12.8	< .001
Gender, n (%)					< .001
Male	3081 (51.2)	760 (37.9)	989 (49.3)	1332 (66.4)	
Female	2937 (48.8)	1246 (62.1)	1017 (50.7)	674 (33.6)	
Race, n (%)					< .001
Mexican American	1239 (20.6)	442 (22)	394 (19.6)	403 (20.1)	
Other Hispanic	234 (3.9)	86 (4.3)	76 (3.8)	72 (3.6)	
Non-Hispanic White	3356 (55.8)	962 (48)	1177 (58.7)	1217 (60.7)	
Non-Hispanic Black	1016 (16.9)	454 (22.6)	295 (14.7)	267 (13.3)	
Other races	173 (2.9)	62 (3.1)	64 (3.2)	47 (2.3)	
Education level, n (%)					< .001
< high school	1013 (16.8)	454 (22.6)	303 (15.1)	256 (12.8)	
High school diploma or GED	2331 (38.7)	832 (41.5)	792 (39.5)	707 (35.2)	
> high school	2674 (44.4)	720 (35.9)	911 (45.4)	1043 (52)	
MS, n (%)					< .001
Married/Living with partner	4004 (66.5)	1195 (59.6)	1356 (67.6)	1453 (72.4)	
Widowed/Divorced/Separated	1679 (27.9)	701 (34.9)	545 (27.2)	433 (21.6)	
Never married	335 (5.6)	110 (5.5)	105 (5.2)	120 (6)	
PIR, n (%)					< .001
Low income	1497 (24.9)	655 (32.7)	453 (22.6)	389 (19.4)	
Medium income	2285 (38.0)	794 (39.6)	792 (39.5)	699 (34.8)	
High income	2236 (37.2)	557 (27.8)	761 (37.9)	918 (45.8)	
Physical activity, n (%)					< .001
Sedentary	2708 (45.0)	1085 (54.1)	856 (42.7)	767 (38.2)	
Moderate	1909 (31.7)	578 (28.8)	694 (34.6)	637 (31.8)	
Vigorous	1401 (23.3)	343 (17.1)	456 (22.7)	602 (30)	
BMI, kg/m^2^	28.5 ± 5.6	28.5 ± 5.7	28.4 ± 5.4	28.5 ± 5.6	.846
Total cholesterol, mg/dL	209.2 ± 41.6	210.4 ± 42.4	208.9 ± 40.1	208.3 ± 42.1	.249
HbA1c, %	5.8 ± 1.1	5.8 ± 1.2	5.8 ± 1.1	5.7 ± 1.1	.015
CRP, mg/dL	0.2 (0.1–0.5)	0.3 (0.1–0.6)	0.2 (0.1–0.5)	0.2 (0.1–0.4)	< .001
Hypertension, n (%)					< .001
No	3859 (64.1)	1216 (60.6)	1276 (63.6)	1367 (68.1)	
Yes	2159 (35.9)	790 (39.4)	730 (36.4)	639 (31.9)	
Diabetes, n (%)					.127
No	5246 (87.2)	1727 (86.1)	1749 (87.2)	1770 (88.2)	
Yes	772 (12.8)	279 (13.9)	257 (12.8)	236 (11.8)	
CVD, n (%)					< .001
No	5093 (84.6)	1651 (82.3)	1703 (84.9)	1739 (86.7)	
Yes	925 (15.4)	355 (17.7)	303 (15.1)	267 (13.3)	
Smoking, n (%)					< .001
Never	2788 (46.3)	924 (46.1)	962 (48)	902 (45)	
Former	2079 (34.5)	631 (31.5)	691 (34.4)	757 (37.7)	
Current	1151 (19.1)	451 (22.5)	353 (17.6)	347 (17.3)	
PAD, n (%)					< .001
No	5660 (94.1)	1842 (91.8)	1906 (95)	1912 (95.3)	
Yes	358 (5.9)	164 (8.2)	100 (5)	94 (4.7)	

T represents tertiles; values are given as mean ± standard deviation or numbers and percentages.

BMI = body mass index, CDAI = composite dietary antioxidant index, CRP = C-reactive protein, CVD = cardiovascular disease, GED = general educational development, HbA1c = hemoglobin A1c, MS = marital status, n = number of participants, PAD = peripheral arterial disease, PIR = poverty-to-income ratio.

### 3.2. Association of CDAI with PAD

Univariate analysis identified multiple factors significantly associated with PAD, including older age, race, education, MS, income, physical activity, CRP, HbA1c, and histories of hypertension, diabetes, and CVD (Table 2). In the fully adjusted multivariate logistic regression model (Model 4, Table 3), each unit increase in the continuous CDAI score was associated with a 4% reduction in the odds of PAD (OR = 0.96; 95% CI = 0.92–1.00; *P* = .033). When analyzed categorically, participants in the T2 tertile had 26% lower odds of PAD compared to the T1 reference group (OR = 0.74; 95% CI = 0.56–0.98; *P* = .037). The T3 group also showed a nonsignificant trend toward lower odds (OR = 0.93; 95% CI = 0.69–1.24; *P* = .611). Table 4 presents the daily intake of each CDAI component and their univariate associations with PAD; higher intakes of selenium, magnesium, zinc, vitamin C, and vitamin E were each associated with significantly lower odds of PAD, while vitamin A showed a nonsignificant inverse trend (OR = 0.869; 95% CI = 0.736–1.025).

**Table 2:** Association of covariates and PAD risk.

Variable	OR (95% CI)	*P* value
Age (yrs)	1.08 (1.07–1.09)	< .001
Gender, n (%)		
Male	1 (reference)	
Female	1.02 (0.82–1.26)	.889
Race, n (%)		
Mexican American	1 (reference)	
Other Hispanic	0.81 (0.4–1.67)	.575
Non-Hispanic White	1.31 (0.97–1.77)	.076
Non-Hispanic Black	1.81 (1.28–2.56)	.001
Other races	0.61 (0.24–1.53)	.29
Education level, n (%)		
< high school	1 (reference)	
High school diploma or GED	0.69 (0.53–0.9)	.007
> high school	0.44 (0.33–0.58)	< .001
MS, n (%)		
Married/Living with partner	1 (reference)	
Widowed/Divorced/Separated	2.01 (1.61–2.5)	< .001
Never married	0.61 (0.32–1.16)	.13
PIR, n (%)		
Low income	1 (reference)	
Medium income	0.8 (0.63–1.02)	.076
High income	0.41 (0.31–0.55)	< .001
Physical activity, n (%)		
Sedentary	1 (reference)	
Moderate	0.61 (0.48–0.77)	< .001
Vigorous	0.28 (0.2–0.41)	< .001
BMI, kg/m^2^	0.99 (0.97–1.01)	.186
Total cholesterol, mg/dL	1 (1–1)	.096
CRP, mg/dL	1.23 (1.13–1.34)	< .001
HbA1c, %	1.21 (1.13–1.3)	< .001
Hypertension, n (%)		
No	1 (reference)	
Yes	2.84 (2.28–3.53)	<0.001
Diabetes, n (%)		
No	1 (reference)	
Yes	2.45 (1.91–3.15)	< .001
CVD, n (%)		
No	1 (reference)	
Yes	3.21 (2.55–4.05)	< .001
Smoking, n (%)		
Never	1 (reference)	
Former	1.92 (1.49–2.46)	< .001
Current	1.92 (1.44–2.56)	< .001
CDAI	0.9 (0.87–0.93)	< .001

BMI = body mass index, CDAI = composite dietary antioxidant index, CRP = C-reactive protein, CVD = cardiovascular disease, GED = general educational development, HbA1c = hemoglobin A1c, MS = marital status, n = number of participants, PAD = peripheral arterial disease, PIR = poverty-to-income ratio.

**Table 3:** Association between CDAI and PAD.

CDAI	Model 1		Model 2		Model 3		Model 4	
	OR (95% CI)	*P* value	OR (95% CI)	*P* value	OR (95% CI)	*P* value	OR (95% CI)	*P* value
Continuous	0.9 (0.87–0.93)	< .001	0.95 (0.91–0.98)	.004	0.95 (0.91–0.98)	.005	0.96 (0.92–1)	.033
Categorical								
T1 (< −7.29)	Ref		Ref		Ref		Ref	
T2 (−1.94–0.78)	0.59 (0.46–0.76)	< .001	0.7 (0.53–0.91)	.009	0.84 (0.63–1.12)	.23	0.74 (0.56–0.98)	.037
T3 (0.78–39.73)	0.55 (0.43–0.72)	< .001	0.82 (0.61–1.09)	.168	0.9 (0.77–1.04)	.147	0.93 (0.69–1.24)	.611

Model 1 adjusted for none.

Model 2 adjusted for age, gender, race, education level, MS, PIR, physical activity.

Model 3 adjusted for age, gender, race, education level, MS, PIR, physical activity, BMI, total cholesterol, CRP, HbA1c.

Model 4 adjusted for all covariates.

T represents tertiles.

BMI = body mass index, CDAI = composite dietary antioxidant index, CI = confidence interval, CRP = C-reactive protein, HbA1c = hemoglobin A1c, MS = marital status, n = number of participants, OR = odds ratio, PAD = peripheral arterial disease, PIR = poverty-to-income ratio, Ref = reference.

**Table 4:** Intake of CDAI components and their univariate association with PAD.

Component	Overall (n = 6018)	PAD (n = 358)	Non-PAD (n = 5660)	*P* value*	OR (95% CI)	*P* value
Selenium (µg)	102.37 ± 63.85	88.63 ± 47.34	103.24 ± 64.65	< .001	0.995 (0.993–0.997)	< .001
Magnesium (mg)	271.02 ± 138.03	234.36 ± 114.38	273.34 ± 139.07	< .001	0.997 (0.997–0.998)	< .001
Zinc (mg)	11.06 ± 8.65	9.43 ± 6.14	11.17 ± 8.78	< .001	0.960 (0.942–0.979)	< .001
Vitamin A (mg)	0.71 ± 1.07	0.63 ± 0.66	0.72 ± 1.09	.018	0.869 (0.736–1.025)	.095
Vitamin C (mg)	95.14 ± 99.00	80.68 ± 75.37	96.05 ± 100.24	.010	0.998 (0.997–0.999)	.004
Vitamin E (mg)	7.17 ± 5.80	5.72 ± 4.48	7.26 ± 5.86	< .001	0.927 (0.900–0.954)	< .001

Values are presented as mean ± standard deviation.

CDAI = composite dietary antioxidant index, CI = confidence interval, n = number of participants, OR = odds ratio, PAD = peripheral arterial disease.

* *P* values compare PAD and non-PAD groups by the Mann–Whitney *U* test. ORs (95% CIs) were estimated by univariate logistic regression per unit increase in each component.

**Figure 2.**
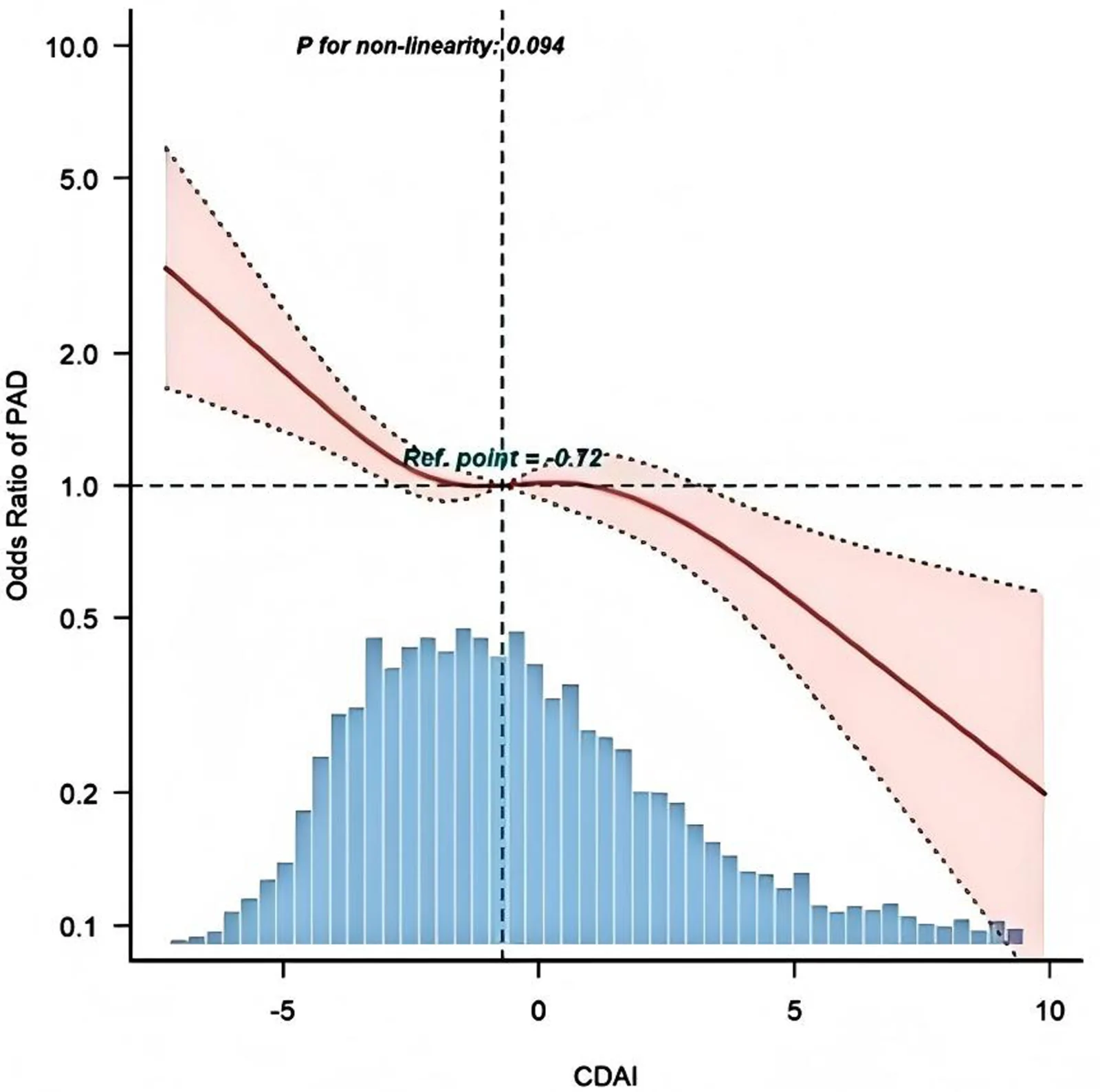
Fitted curve of the association between CDAI and PAD. CDAI = composite dietary antioxidant index, PAD = peripheral artery disease.

Restricted cubic spline analysis (Fig. 2) visually confirmed an inverse, nonlinear relationship between CDAI and PAD risk (*P* for nonlinearity = .094). The risk curve declined until reaching a CDAI value of approximately −0.72, beyond which the risk plateaued, suggesting a potential threshold effect.

### 3.3. Subgroup analyses

**Figure 3.**
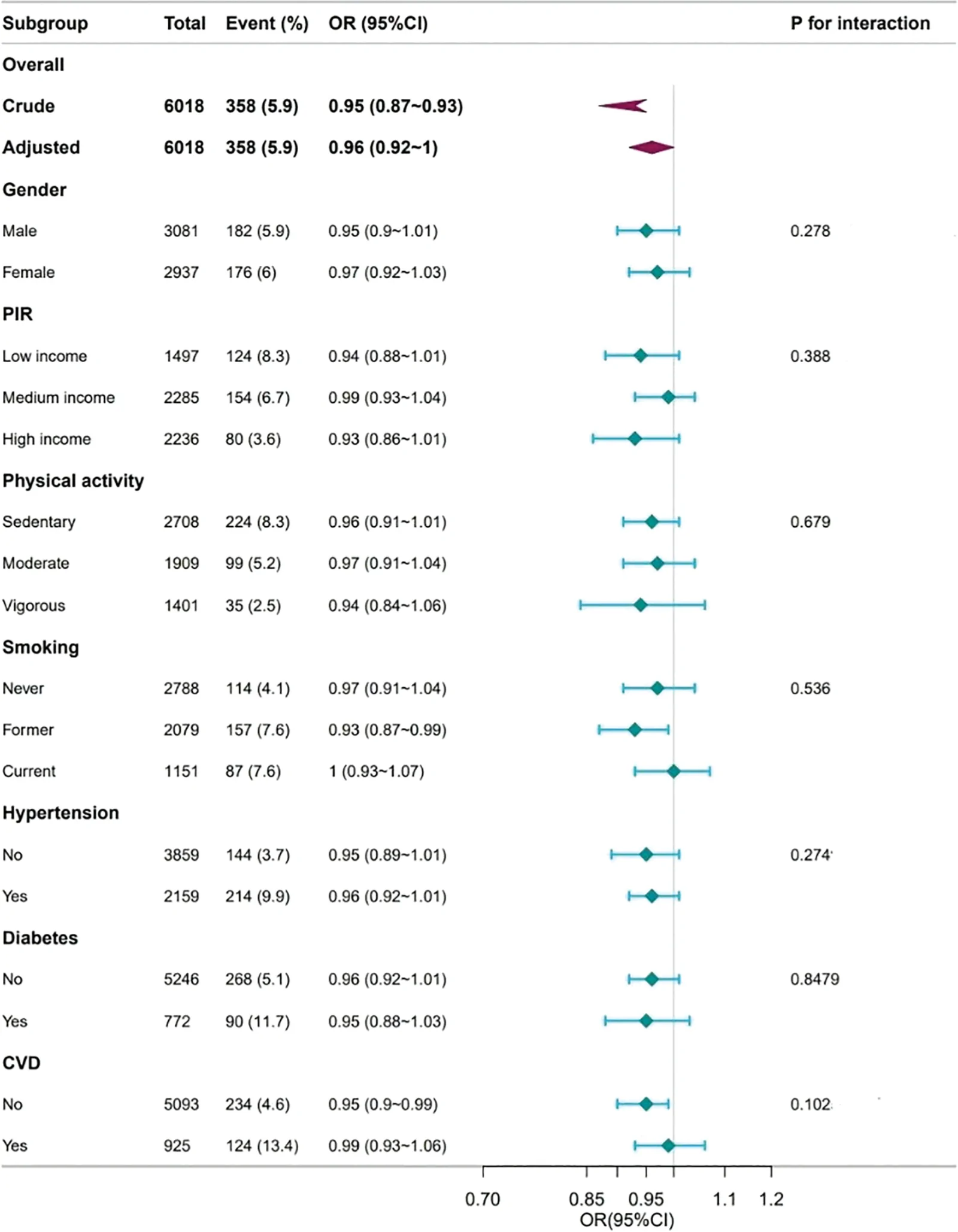
Effect of CDAI on PAD in each subgroup. CDAI = composite dietary antioxidant index, CI = confidence interval, CVD = cardiovascular disease, OR = odds ratio, PAD = peripheral artery disease, PIR = poverty-to-income ratio.

Stratified analyses were performed across subgroups defined by sex, hypertension, diabetes, CVD history, PIR, physical activity, and smoking status (Fig. 3). The inverse association between CDAI and PAD was consistent in direction and magnitude across all subgroups. No statistically significant interactions were observed (all *P* for interaction > .05), indicating that the relationship was not modified by these common PAD risk factors.

## 4. Discussion

In this large, cross-sectional study of US adults, we observed a significant inverse association between CDAI and the prevalence of PAD. This relationship persisted after extensive adjustment for demographic, lifestyle, and clinical confounders, suggesting that a diet rich in antioxidants may be protective against PAD. Our findings contribute to the expanding literature on the cardioprotective role of dietary antioxidants, with specific implications for PAD pathophysiology.

The observed protective effect of a higher CDAI aligns with previous reports on individual antioxidants. For instance, Naqvi et al^[19]^ documented inverse associations between PAD and intakes of vitamins A, C, and E, independent of traditional risk factors. Similarly, an analysis of NHANES data found protective effects for vitamin A (OR = 0.79), vitamin C (OR = 0.84), and vitamin E (OR = 0.78).^[18]^ Antonelli-Incalzi et al^[17]^ reported that vitamin E intake ≥ 7.726 mg/d was linked to a significantly reduced PAD risk (OR = 0.37). The Rotterdam study^[21]^ noted sex-specific associations, with vitamin E intake inversely related to PAD in men and vitamin C in women. Recent evidence also supports a role for magnesium,^[22]^ while preclinical studies suggest zinc and selenium protect against atherosclerotic processes.^[38]^ A meta-analysis by Zhang et al^[39]^ indicated an inverse relationship between selenium status and CVD risk within a specific concentration range.

Our study extends this evidence by demonstrating that a composite measure of antioxidant intake is similarly associated with a lower likelihood of PAD. In our fully adjusted model, each unit increase in CDAI corresponded to a 4% reduction in PAD odds. Participants with moderate antioxidant intake (T2) had a significantly 26% lower odds of PAD compared to the lowest consumers (T1). The nonsignificant effect in the highest tertile (T3) may suggest a plateauing benefit, consistent with our spline analysis, and warrants further investigation. The consistency of this association across diverse subgroups strengthens the robustness of our findings.

The observed inverse association may be explained by the capacity of dietary antioxidants to counteract oxidative stress and inflammatory processes, both recognized as pivotal drivers in the pathogenesis of atherosclerosis.^[40]^ Antioxidants like vitamins C and E scavenge reactive oxygen species, reducing oxidative damage to vascular components.^[13,15,18]^ Vitamin A contributes to endothelial integrity and immune function.^[19]^ The trace elements in CDAI also play vital roles: magnesium regulates vascular tone and inflammation,^[22,41]^ while zinc and selenium are essential for maintaining redox homeostasis.^[21,38,42,43]^ The CDAI likely captures the synergistic action of these nutrients, offering a more holistic measure of antioxidant defense.

A key strength of our study is the use of CDAI, which provides an integrated assessment of dietary antioxidant capacity beyond single-nutrient analyses. The use of a large, nationally representative sample and comprehensive adjustment for confounders further strengthens our conclusions.

Several limitations warrant consideration. The cross-sectional design precludes causal inference. Although we adjusted for numerous confounders, residual confounding from unmeasured factors like detailed dietary patterns or genetic predisposition cannot be ruled out. The use of a single 24-hour recall may not fully represent habitual intake. Furthermore, our analysis did not incorporate sampling weights, which may affect the generalizability of the prevalence estimates, though it likely has less impact on internal validity for exposure-disease associations. Future prospective cohorts and randomized trials are needed to establish causality and define optimal intake levels. Integrating biomarker assessments and experimental models will help elucidate the precise biological pathways.

## 5. Conclusions

In summary, this study demonstrates that a higher CDAI is independently associated with a lower prevalence of PAD among US adults. These results underscore the potential importance of a diet rich in diverse antioxidants for PAD prevention. Healthcare providers should consider dietary antioxidant quality when advising patients at risk for PAD. Further longitudinal and interventional studies are needed to confirm these findings and translate them into clinical practice.

## Acknowledgments

We express our gratitude to Jie Liu from the Department of Vascular and Endovascular Surgery at Chinese PLA General Hospital for providing valuable assistance in statistical analysis, study design consultations, and offering insightful feedback on the manuscript. Jie Liu has given permission to be named in the acknowledgments.

## Author contributions

**Conceptualization:** Qiang Liu, Jianjun Shi.

**Data curation:** Xing Wu.

**Formal analysis:** Jun Yan.

**Funding acquisition:** Jianjun Shi.

**Investigation:** Yigang He.

**Methodology:** Jun Yan.

**Project administration:** Yigang He.

**Resources:** Yigang He.

**Software:** Xing Wu.

**Supervision:** Yun Wang, Jianjun Shi.

**Validation:** Yun Wang.

**Writing – original draft:** Qiang Liu, Xing Wu.

**Writing – review & editing:** Jianjun Shi.
